# The validity of current implantable cardioverter-defibrillator guidelines in a real-world population of adults with congenital heart disease: A single-center experience

**DOI:** 10.1016/j.ijcchd.2022.100355

**Published:** 2022-03-17

**Authors:** Satoshi Kawada, Praloy Chakraborty, Jared Nanthakumar, Lisa Albertini, Erwin N. Oechslin, Susan Lucy Roche, Candice Silversides, Rachel M. Wald, Eugene Downar, Louise Harris, Lorna Swan, Rafael Alonso-Gonzalez, Sara Thorne, Kumaraswamy Nanthakumar, Blandine Mondésert, Paul Khairy, Krishnakumar Nair

**Affiliations:** a University Health Network Toronto, Peter Munk Cardiac Centre, and University of Toronto, Toronto, Ontario, Canada; b Adult Congenital Heart Disease Center, Department of Medicine, Montreal Heart Institute, University of Montreal, Montreal, Canada

**Keywords:** Implantable cardioverter defibrillator, Sudden cardiac death, Adult congenital heart disease

## Abstract

**Aims:**

Sudden cardiac death (SCD) is a major cause of mortality in adults with congenital heart disease (ACHD). The role of implantable cardioverter-defibrillator (ICDs) in preventing SCD has been established, however, robust, clinical evidence-based guidelines are lacking in ACHD. The aim of this study was to evaluate the ICD guidelines in ACHD patients.

**Methods and Results:**

A total of 131 ACHD patients (male: n = 96 (73.3%), mean age: 42.8 ± 14.7 years, mean follow-up: 40.9 ± 28.3 months) undergoing ICDs implantation between 2010 and 2017 were reviewed. Sixty-nine patients (52.6%) received ICDs for a primary prevention indication. 122 (93.3%) patients had congenital heart disease of moderate to severe complexity. CRT-D (implantable cardiac resynchronization defibrillator) was implanted in 55 (42.0%) patients. During follow-up, 23 patients (17.6%) received appropriate ICD therapy. According to the current guideline (PACES/HRS 2014), 84 (64.1%), 8 (6.1%), and 39 (29.8%) could be classified as Class Ⅰ, Class Ⅱa, and Class Ⅱb indication, respectively. Compared to patients with Class Ⅱa and IIb indication, those with Class Ⅰ indication received more appropriate therapy (P = 0.030). Multivariate analysis showed that age (per 10-years decrease; P = 0.015, HR 1.254 CI; 1.045–1.505) and creatinine (per 100-μmol/L increase; P = 0.019, HR 1.555 CI; 1.076–2.247) were associated with appropriate therapy.

**Conclusion:**

Implantation of ICDs for preventing SCD based on current guidelines is reasonable. For patients with a borderline indication, younger age and renal dysfunction may aid in the selection of ICDs candidates.

## Highlights

• Translational aspect of the work.
• Our study demonstrated that the frequency of appropriate ICD therapy events increases with the strength of recommendation of the guidelines.
• Younger age and renal dysfunction were associated with appropriate therapy.

## 1 Introduction

Owing to earlier diagnosis and improvement in surgical interventions, an increasing number of adult congenital heart disease (ACHD) patients are surviving to adulthood. Incidence of sudden cardiac death (SCD) is 25–100 times greater in this population [1] and accounts for 19–26% of mortality in this group [1,2]. Ventricular arrhythmias are responsible for 80% of SCD. Implantable cardioverter defibrillators (ICDs) are proved to be effective in preventing SCD in ACHD patients. ICD implantation is currently performed based on international guidelines (Pediatric and Congenital Electrophysiology Society [PACES]/Heart Rhythm Society [HRS] 2014 and European Heart Rhythm Association [EHRA] 2018). The number of ICDs implanted is progressively increasing [3,4] and the current guidelines for such implantations are based on expert opinion, data extrapolated from patients with acquired heart diseases, and limited retrospective data from tetralogy of Fallot (TOF) patients. No randomized control data are available due to a relatively smaller number of patients with diverse pathologies [5]. However, substrate characteristics in ACHD patients differ from those with acquired heart disease. For example, due to repeated surgery, the ventricular scar burden in ACHD patients may be disproportionately high and may not correlate with the systemic ventricular function, the deciding parameter for primary prevention in acquired heart disease. Some ACHD patients have significantly abnormal substrate only in the sub-pulmonary ventricle (right ventricle: RV) with the normal function of the systemic subaortic ventricle (left ventricle: LV). The RV myocardial architecture and orientation of the fibers are different from LV musculature, and it is difficult to assess the arrhythmic risk of isolated RV dysfunction. The optimal cutoff value for ventricular function for risk stratification has not been established [3,4]. Other factors such as syncope, the complexity of congenital heart disease, ventricular dysfunction with NYHA I, and ventricular tachycardia (VT)/ventricular fibrillation (VF) inducible at electrophysiology testing (EPS) are also considered in risk assessment of SCD. However, the role of the above factors in risk assessment is not based on robust clinical data. Moreover, complex anatomy leads to higher procedural and lead-related complications in this group of patients [8]. Additionally, the high burden of atrial arrhythmia makes these patients more vulnerable to inappropriate ICD therapy [6]. Considering the difficulties in conducting a prospective study in this group of patients, we sought to evaluate the appropriateness of guidelines by analysis of long-term follow-up data when patients were classified retrospectively as per guideline recommendations. The aim of this study was to evaluate the efficacy of current ICD guidelines and identify the risk factors for ventricular arrhythmia in ACHD patients.

## 2 Methods

### 2.1 Patient population and study design

Patients with ACHD undergoing initial ICD/CRT-D (implantable cardiac resynchronization therapy defibrillator)/SICD (subcutaneous ICD) placement at Toronto General Hospital from 2010 to 2017 were reviewed. After obtaining approval from the University Health Network Research Ethics Board, medical records of all patients were examined for baseline characteristics, clinical presentation, the indication of device implantation and procedural details.

### 2.2 Classification of indications of ICD

All patients were retrospectively categorized into 3 groups (Class Ⅰ, Class Ⅱa and Class Ⅱb or recommended/indicated and may be recommended/indicated) according to the current guidelines [4,5]. All patients undergoing ICD implantation for secondary preventions or primary prevention for systemic LV function <35% with NYHA II-III were classified as Class I indication (or recommended/indicated). Higher-risk patients with TOF with LVEF>35% were classified as Class Ⅱa. Patients with single or systemic RVEF<35% with additional high-risk features or unexplained syncope with moderate/complex anatomy, or those waiting for heart transplantation were classified as Class Ⅱb (or maybe used or recommended). (Table 1, Table 2). Two independent electrophysiologists (S.K and L.A), after reviewing medical records, classified the patients as per indications.

**Table 1:** Baseline patient characteristics of total patients and 3 groups according to the PACES/HRS 2014 guideline.

	Total (N = 131)	Class Ⅰ (N = 84)	Class Ⅱa (N = 8)	Class Ⅱb (N = 39)	P value
Age (years)	42.8 ± 14.7	43.2 ± 14.4	46.5 ± 16.6	41.2 ± 15.4	0.610
Sex (Male)	96 (73.3%)	62 (73.8%)	5 (62.5%)	29 (74.4%)	0.775
Body mass index (kg/m2)	26.9 ± 5.6	27.1 ± 5.9	23.6 ± 5.2	27.2 ± 5.0	0.282
NYHA Ⅱ-Ⅲ (n, %)	53 (74.7%)	32 (76.2%)	3 (75.0%)	18 (72.0%)	0.930
Complex anatomy (%)	61 (46.6%)	29 (34.5%)	0 (0%)	32 (82.0%)	N/A
Primary prevention (%)	69 (52.7%)	22 (26.2%)	8 (100%)	39 (100%)	N/A
Biventricular pacing system (CRT-D) (%)	55 (42.0%)	28 (33.3%)	4 (50.0%)	23 (59.0%)	0.025
Total follow-up periods (month)	40.9 ± 28.3	44.9 ± 29.8	39.5 ± 25.4	32.5 ± 23.8	0.073
BNP (pg/ml)	249.5 (127.7–546.9)	256.2 (137.1–580.1)	470.0 (125.2–807.1)	237.5 (75.0–465.8)	0.829
Creatinine (umol/L)	98.4 ± 70.4	102.4 ± 82.9	118.3 ± 92.7	86.6 ± 22.4	0.398
Albumin (g/dl)	38.7 ± 5.1	37.7 ± 4.7	42.4 ± 2.4	40.3 ± 5.7	0.949
QRS duration (ms)	164.0 ± 39.0	167.3 ± 41.8	173.4 ± 17.1	155.7 ± 35.0	0.272
Echocardiogram					
Systemic ventricular EF (%)	40.4 ± 16.1	42.3 ± 16.5	45.1 ± 17.5	33.2 ± 12.5	0.027
Subpumonary ventricular FAC (%)	26.5 ± 9.9	26.6 ± 9.9	39.8 ± 3.4	22.3 ± 13.4	0.456
Subpulmonary systemic ventricular pressure (mmHg)	45.5 ± 14.4	44.7 ± 14.6	53.5 ± 16.4	44.9 ± 13.0	0.367
Previous VT ablation (%)	9 (6.9%)	9 (10.7%)	0 (0.0%)	0 (0.0%)	0.067
Heart failure admission history (%)	65 (49.6%)	37 (44.0%)	4 (50.0%)	24 (61.5%)	0.196
Previous AT/AF (%)	35 (62.5%)	17 (60.7%)	0 (0.0%)	18 (64.5%)	0.783
Coronary artery disease (%)	7 (5.3%)	5 (6.0%)	0 (0.0%)	2 (5.1%)	0.772
Beta blocker (%)	99 (78.0%)	60 (74.1%)	8 (100%)	31 (18.4%)	0.196
ACEI/ARB (%)	57 (44.9%)	28 (34.6%)	3 (37.5%)	26 (68.4%)	0.002
Amiodarone (%)	39 (30.7%)	28 (34.6%)	1 (12.5%)	10 (26.3%)	0.340

ACEI, angiotensin-converting enzyme inhibitor; ARB, angiotensin receptor blocker; AF, atrial fibrillation; AT, atrial tachycardia; BNP, brain natriuretic peptide; EF, ejection fraction; EPS, electrophysiology test; FAC, fractional area change, NYHA, new york heart association; VT, ventricular tachycardia.

**Table 2:** Patient distribution according to the PACES/HRS 2014 and EHRA 2018 guidelines.

	PACES/HRS		EHRA	Type of prevention	Present study (N = 131)		
	Class	Level of evidence			Patients distribution (N = 131)	Appropriate ICD therapy (N = 23)	Inappropriate ICD therapy (N = 13)
1. Survivors of cardiac arrest	Ⅰ	B	Recommended/indicated	Secondary prevention	23 (17.6%)	6 (26.1%)	4 (30.8%)
2. Spontaneous sustained VT	Ⅰ	B			39 (29.8%)	9 (39.1%)	5 (38.4%)
3. Systemic LVEF ≤35%, biventricular physiology, NYHA Ⅱ-Ⅲ	Ⅰ	B		Primary prevention	22 (16.8%)	5 (21.7%)	1 (7.7%)
1. Tetralogy of Fallot with multiple risk factors (LV systolic or diastolic dysfunction, NSVT, QRS duration ≥180 ms, extensive RV scarring, positive EPS)	Ⅱa	B	May be used or recommended		8 (6.1%)	1 (4.3%)	1 (7.7%)
1. Syncope of unknown origin with positive EPS	Ⅱb	B			3 (2.3%)	0 (0.0%)	0 (0.0%)
2. Single or systemic RV EF<35% with additional risk (complex ventricular tachycardia, unexplained syncope, NYHAⅡ-Ⅲ, QRS duration ≥140 ms, or severe systemic AV valve regurtitation),	Ⅱb	C			24 (18.8%)	1 (4.3%)	0 (0.0%)
3. Awaiting heart transplantation	Ⅱb	C			1 (0.8%)	0 (0.0%)	0 (0.0%)
4. Syncope and moderate or complex anatomy	Ⅱb	C			8 (6.3%)	1 (4.3%)	2 (15.4%)
5. Systemic ventricular EF<35% with NYHA1	Ⅱb	C	N/A		3 (2.3%)	0 (0.0%)	0 (0.0%)

### 2.3 ICD programming

Device programming was performed as per the institution protocols for primary and secondary prevention. For primary prevention, VT detection was programmed to/at rates greater than 196 beats/min (bpm) with the delivery of 3 predefined sequences of anti-tachycardia pacing (ATP) followed by maximal energy shocks. VF detection was activated above 214 bpm with maximal energy shock therapy programmed, preceded by 1 ATP during charging. Secondary prevention programming was individualized, and parameters were tailored according to the cycle length of clinical ventricular arrhythmias. In patients with recorded clinical VT, the VT zone was programmed with 10–20 bpm under clinical VT. For secondary prevention in patients with unknown VT cycle length, VT detection was programmed at rates higher than 187 bpm with the delivery of 3 predefined sequences of ATP followed by maximal energy shock. VF detection was activated above 230 bpm with ATP while charging, followed by maximal energy shock.

### 2.4 Endpoints

The primary endpoint of this study was the development of appropriate ICD therapy (either shock or ATP) after ICDs implantation. The secondary endpoint of this study was any cardiac events, including heart failure (HF) hospitalization and major cardiac events (MACE). MACE was defined as death from any cause and heart transplantation.

### 2.5 Patient evaluation and follow-up

All patients were evaluated in the outpatient clinic at 1 month after implantation. Patients were seen in clinical follow-up at 3–6 months intervals. Clinical evaluation and device testing were carried out at each follow-up visit. Appropriate therapy was defined as those delivered for ventricular arrhythmias such as VT and VF. Therapies were considered as inappropriate when delivered for a rhythm other than VT or VF.

### 2.6 Statistical analysis

Data are presented as mean ± SD or median (interquartile range: IQR) for continuous variables, depending on the Shapiro-Wilk test for normality. Student's t-test or Mann-Whitney *U* was used to compare continuous variables between groups. Categorical variables are presented as absolute values and proportions (%). The Pearson Chi-square test or Fisher exact test was used to compare categorical variables between groups. We calculated the hazard ratio (HR) with a 95% confidence interval (CI) for each variable that was significantly associated with the occurrence of appropriate ICD therapy. Survival and cumulative hazards were calculated using the Kaplan-Meier method. Differences between survival curves were compared using the log-rank test. All tests were performed using SPSS 26.0 software Mac OS version (IBM Corp., Armonk, New York, USA). Values of P < 0.05 were considered statistically significant.

## 3 Results

### 3.1 Patients’ characteristics and diagnosis

**Fig. 1:**
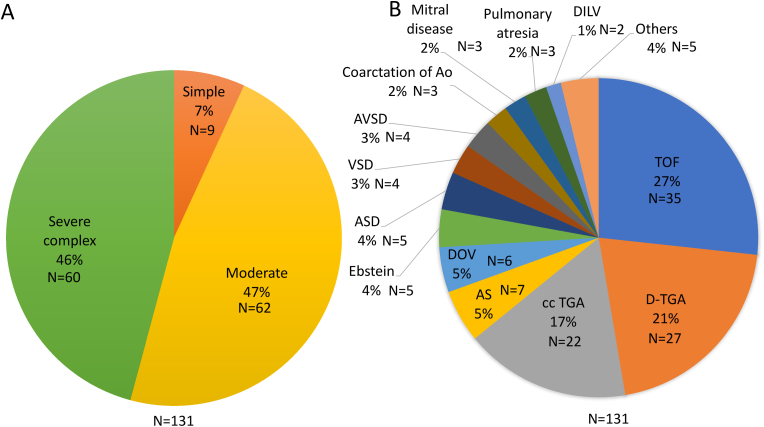
The prevalence of the individual diagnosis. A. Classification according to the anatomical complexity., B. Anatomical distribution. Ao, Aorta; AS, congenital aortic stenosis; ASD, atrial septal defect; AVSD, atrioventricular septum defect; cc TGA, congenitally corrected transposition of the great arteries; DILV, double inlet left ventricle; DOV, double orifice ventricle; D-TGA, d-transposition of the great arteries; , TOF, tetralogy of Fallot; VSD, ventricular septal defect; Others include unrepaired ASD, unrepaired VSD, TAPVC (total anomalous pulmonary venous return), VSD with right coronary cusp prolapse and Fontan (mitral atresia).

A total of 131 ACHD patients who underwent ICDs implantation at Toronto general hospital between 2010 and 2017 were reviewed (male n = 96 [73.3%]; mean age 42.8 ± 14.7 years). The mean follow-up period was 40.9 ± 28.3 months. 55 (42.0%) patients received biventricular pacing/defibrillation system. Epicardial implantation was performed in 2, and all other patients received a transvenous (n = 124) or subcutaneous (n = 5) ICD system. More than one-third of the patients (47 patients; 35.9%) were upgraded from a pacemaker or cardiac resynchronization therapy pacing (CRT-P) to ICD/CRT-D. Mean systemic ventricular ejection fraction (EF) and sub-pulmonary ventricular fractional area change (FAC) obtained by echocardiogram were 40.4 ± 16.1 and 26.5 ± 9.9%, respectively. Almost all of the patients were of moderate to severe complex anatomy (n = 122, 93.1%) [3]. Thirty-seven patients underwent EPS before ICD implantation. Of them, VT/VF was induced in 24 patients and 9 patients underwent VT ablation. (Table 1). The most common diagnosis was TOF with 35 patients (26.7%) followed by d-transposition of the great arteries (D-TGA) (n = 27, 20.6%) and congenitally corrected transposition of the great arteries (cc TGA) (n = 22, 16.8%) (Fig. 1). Patient characteristics according to the individual diagnosis are shown in supplement Table 2.

Patients were classified into 3 categories with respect to current guidelines; Class I (n = 84, 64.1%), Class Ⅱa (n = 8, 6.1%) and Class Ⅱb (n = 39, 29.8%) or recommended/indicated (n = 84, 64.1%) and may be used/recommended (n = 44, 33.6%) [3,4]. The PACE/HRS 2014 guideline was applicable to all patients, however, 3 (2.3%) patients (systemic ventricular EF <35% with NYHAⅠ) did not fulfill criteria for ICD implantation according to EHRA 2018 guideline. There was a significant difference in the prevalence of CRT-D (P = 0.025) and systemic ventricular EF (P = 0.027) among groups.

### 3.2 ICD therapy events and ICD indications

A total of 33 patients (25.2%) received ICD therapy during follow-up. Of them, 20 (15.3%) patients received appropriate ICD therapy, and 10 (7.6%) patients experienced inappropriate ICD therapy, and only 3 (2.3%) patients had both appropriate and inappropriate ICD therapy (Table 2). The annual incidence of appropriate ICD therapy was 6.0%/year (7.1%/year for secondary prevention, 4.6%/year for primary prevention). Appropriate ICD therapies were delivered due to VF in 3 (13.0%) patients and VT in 20 (87.0%) patients. Of them, 9 patients had both shock and ATP, and 10 patients received only shock. All ventricular arrhythmias were terminated successfully via ATP or shock. Patients with appropriate ICD therapy (n = 23, 17.6%) were significantly younger (34.9 ± 12.9 vs. 44.5 ± 14.6, P = 0.003) with higher prevalence of secondary prevention (65.2% vs. 42.7%, P = 0.049) and Class Ⅰ indications (87.0% vs. 59.3%, P = 0.010) compared to the patients without appropriate therapy (n = 108, 82.4%). Patient characteristics with or without appropriate ICD therapy are described in supplement Table 1.

The annual incidence of inappropriate ICD therapy was 3.1%/year. Inappropriate ICD therapies were caused by supraventricular tachycardia in 9 (69.2%) patients, T wave over-sensing in 1 (7.7%) patient, and sinus tachycardia in 3 (23.1%) patients. Kaplan-Meier analysis of time to first appropriate or inappropriate ICD therapy is demonstrated in Supplement Figure 1.

Details of ICD indication and therapy events are listed in Table 2. Sixty-nine (52.7%) and 62 (47.3%) patients were implanted with ICDs for primary and secondary prevention, respectively. In patients with primary prevention ICDs, 22 patients (16.8%) received ICD for systemic LV dysfunction and advanced HF symptoms (NYHA II-IV) and 8 patients had TOF with multiple risk factors (Class Ⅱa indication). In the primary prevention group, 39 patients (29.8%) satisfied criteria for a Class Ⅱb indication, including poor single or systemic RV dysfunction, history of syncope, and positive EP study. Of the patients who received secondary prevention ICDs (Class I), 23 patients (17.6%) received ICDs after cardiac arrest (including polymorphic VT and VF) and 39 patients (29.8%) due to sustained monomorphic VT.

**Fig. 2:**
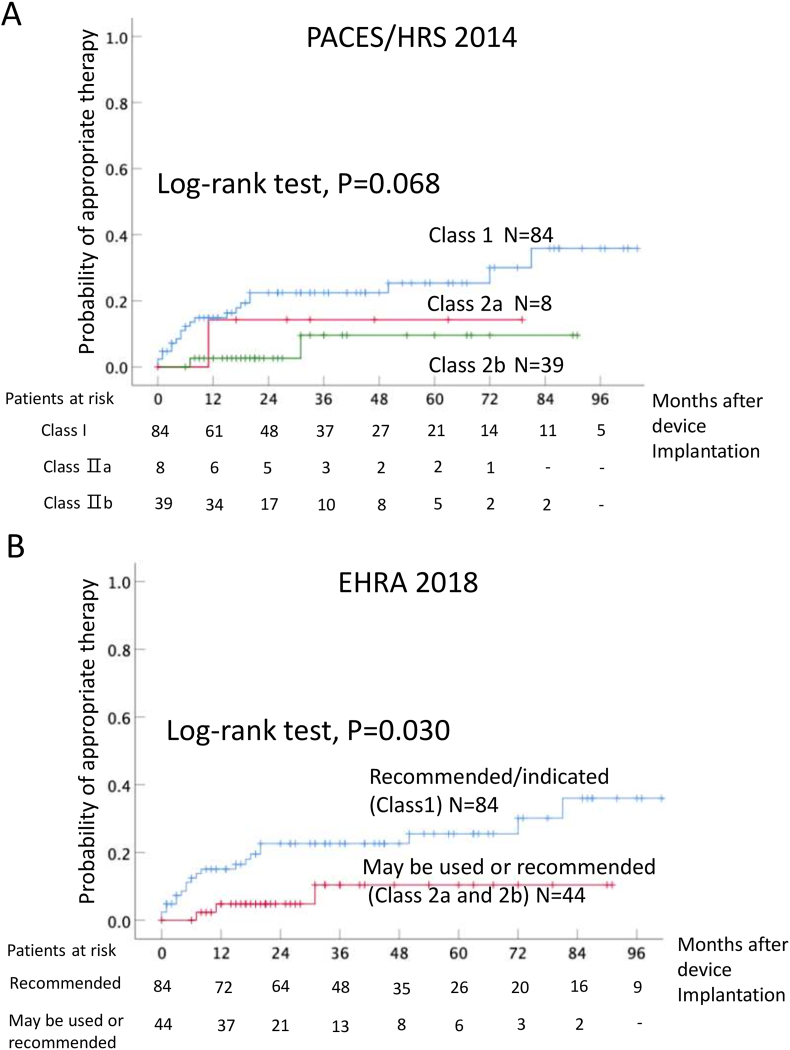
Kaplan-Meier analysis of cumulative appropriate ICD therapy events.A. Cumulative appropriate ICD therapy events according to the PACES/HRS 2014 guideline, B. Cumulative appropriate ICD therapy events according to the EHRA guideline.

During follow-up, 20 patients (23.8%) with a Class Ⅰ indication received appropriate ICD therapy. On the other hand, only 1 patient (4.3%) with a Class Ⅱa indication and 2 patients (8.6%) with a Class Ⅱb indication received appropriate ICD therapy. The annual incidence of appropriate ICD therapy in Class Ⅰ, Class Ⅱa, and Class Ⅱb was 7.6%, 4.3%, and 2.0%/year, respectively. Kaplan-Meier survival curve showed there was no significant difference between 3 groups (P = 0.068) (Fig. 2A), however, patients with Class Ⅰ indication (recommended/indicated) had significantly received more appropriate therapy than Class Ⅱa and Class Ⅱb indication (may be or used or recommendation) (P = 0.030) (Fig. 2B). If data were limited to patients with primary prevention (n = 69), the same trends were obtained (supplement Fig. 2A and 2B).

### 3.3 Outcomes related to appropriate therapy

During follow-up periods of 40.9 ± 28.3 months, 33 patients (25.2%) were admitted to the hospital due to HF. The annual incidence of HF was 8.2%/year. HF developed in eight patients (34.8%) and 25 patients (23.1%) with and without appropriate ICD therapy, respectively. There was no significant difference between the 2 groups (P = 0.704) (supplement Figure 3A).

The annual incidence of MACE was 5.0%/year. In total, 22 patients (16.8%) died or underwent a heart transplant. Of all patients, 12 (9.2%) patients received a heart transplant and 2 (1.5%) patients died due to cardiac arrest after VT storm or atrial tachyardia (AT) with 1:1 rapid ventricular response. Four (3.1%) patients died because of end-stage HF. Details of death in other cases (n = 4) were unknown. No significant difference was seen in Kaplan-Meier analysis between patients with and without appropriate ICD therapy (P = 0.743) (supplement Figure 3B).

### 3.4 Predictors of appropriate ICD therapy

Univariate and multivariate analyses of clinical risk factors related to appropriate ICD therapy are listed in Supplement Table 3. Univariate analysis showed that appropriate ICD therapy was associated with younger age (per 10-years decrease; P = 0.022, HR; 1.172, CI; 1.023–1.344), Class Ⅰ indication (or recommended/indicated) (P = 0.031, HR; 3.79, CI; 1.121–12.81), higher creatinine (per 100-μmol/L increase; P = 0.034, HR; 1.528, CI; 1.032–2.262) and lower albumin (10-μmol/L increase; P = 0.040, HR; 0.392, CI; 0.160–0.959). History of AT/atrial fibriilation (AF) did not achieve significance (P = 0.079). VT ablation prior to ICD implantation was not related to appropriate ICD therapy events (P = 0.612). Systemic and sub-pulmonary ventricular EF was not related to appropriate ICD therapy (P = 0.403 and P = 0.775).

On multivariate analysis, younger age (per 10-years decrease; P = 0.015, HR; 1.254, CI; 1.045–1.505) and higher creatinine (per 100-μmol/L increase; P = 0.019, HR; 1.045, CI 1.007–1.084) were associated with appropriate ICD therapy. Class Ⅰ indication (or recommended/indicated) seemed to be associated with appropriate ICD therapy but did not show statistical significance (P = 0.075, HR; 6.488, CI; 0.828–50.84).

## 4 Discussion

### 4.1 Main findings

Our single-center retrospective study had 3 main findings. First, this study demonstrated that the incidence of appropriate ICD therapy was 17.6% (n = 23) (15 patients with secondary prevention and 8 patients with primary prevention) during a mean follow-up period of 40.9 ± 28.3 months. Second, all ICD recipients fulfilled the criteria for ICD implantation according to PACE/HRS 2014 guidelines. The frequency of appropriate ICD therapy events varied directly with the strength of recommendation of the guidelines. The annual incidence of appropriate ICD therapy in Class Ⅰ, Class Ⅱa, and Class Ⅱb was 7.6%/year, 4.3%/year, and 2.0%/year, respectively. Finally, younger age and renal dysfunction were associated with appropriate ICD therapy.

### 4.2 Indication for ICD therapy in ACHD

In our study, all patients could be assigned according to either PACE/HRS 2014 or EHRA 2018 guidelines, retrospectively (ClassⅠ: n = 84, 64.1%, Class Ⅱa: n = 8, 6.1%, Class Ⅱb: n = 39, 29.8% or recommended/indicated: n = 84, 64.1%, may be used or recommended: n = 44, 33.6%). Although PACES/HRS 2014 and EHRA 2018 guidelines are similar to each other, there are some differences between them. PACES/HRS 2014 guideline assigns a ClassⅡb indication for patients with systemic ventricular EF <35% with NYHAⅠand Class Ⅲ indication for patients with Eisenmenger syndrome; while the EHRA 2018 guideline has not included the above mentioned groups^3 4^. These differences have not been evaluated yet. In our retrospective study, 3 patients did not fulfill the criteria of the EHRA 2018 guideline despite satisfying Class Ⅱb indications of the PACE/HRS 2014 guidelines. Fortunately, they did not develop appropriate ICD therapy during follow-up. Current guidelines also take account of the NYHA functional class for the selection of patients for ICD implantation. However, evaluating precise functional class is often difficult in a clinical setting. Since most of the ACHD patients are accustomed to relatively poor physical activities, they feel that their exercise capacity is often preserved. Therefore, functional class is often comparable to those of the general population [7]. Engelfriet et al. reported that 91% of adult Fontan patients were considered in NYHA Ⅰ-Ⅱ despite a reduction in exercise tolerance [8]. Objective evaluation with cardiopulmonary testing should be considered in borderline patients. Each case should be evaluated in a multidisciplinary meeting with cardiologists and electrophysiologists specializing in ACHD. Further studies are mandatory to fill in the gaps between guidelines and real-world findings.

### 4.3 The incidence of ICD therapy

Our study showed that the annual incidence of appropriate ICD therapy events increased with the level of recommendation of the guidelines, indicating that complying with guidelines is reasonable. Previous studies reported that ACHD patients with ICDs had both appropriate and inappropriate ICD therapy frequently [9]. In this study, the frequency of appropriate ICD therapy is consistent with other studies in patients without ACHD, however, a little lower than other congenital studies [6,10]. One of the reasons is the differences in baseline patient demographics. The most common diagnosis in our study was TOF (n = 35, 27%) followed by d-TGA (n = 27, 21%) and cc TGA (n = 22, 17%), whereas half of the patients were TOF in other studies. Other explanations are the frequent use of beta-blocker (78.0%) and programmed settings of devices [11,12]. It is noteworthy that the majority of the appropriate ICD therapy (87.0%) was related to sustained monomorphic VT and was successfully terminated by shock or ATP. ATP is thought to be highly effective in terminating VT in ACHD patients [13].

### 4.4 Risk factors for appropriate ICD therapy

The identification of patients for primary prevention remains challenging in ACHD patients. Even current guidelines for primary prevention fail to recognize 60–65% of the high-risk group in the ACHD cohort^14^**.** Therefore, detailed evaluation based on not only guidelines but the physicians’ clinical judgment is still needed in decision making, also taking account of the risk factors in each case. Several risk factors for appropriate ICD therapy have been reported [5]. Koyak et al. reported that secondary prevention, coronary artery disease, and non-sustained VT (NSVT) were associated with appropriate ICD therapy [5]. In our study, there was no association between appropriate ICD therapy and these risk factors. This might also be explained by the difference in patient demographics. Compared to their study, our study included more moderate-severe complex anatomy patients (93.0%) with a high prevalence of CRT-D (42.0%) and less prevalence of coronary artery disease (5.3%). NSVT is frequently seen in moderate-severe complex anatomy (up to 60% in patients with TOF) [15], it was also thought to be more common in our study in comparison to previous studies because of the high prevalence of moderate-severe anatomy [5]. QRS prolongation is reported as a risk factor of SCD in TOF patients [16], however, the QRS duration did not correlate with appropriate ICD therapy in our study. Kapel et al. reported that VT ablation was effective in the management of ventricular arrhythmia in patients with TOF [17]. VT ablation was not related to appropriate ICD therapy in our cohort. However, regarding the TOF patients in our study, no patient who underwent VT ablation received appropriate ICD therapy during follow-up. VT ablation before ICD implantation and after the first ICD therapy should be considered in patients with TOF to decrease appropriate ICD therapy.

More than one-third of the patients (n = 47, 35.9%) were upgraded from pacemaker or CRT-P, hence, intrinsic QRS duration could not be evaluated in many cases. On the other hand, our study showed that younger age (per 10-years decrease; P = 0.015, HR; 1.254, CI; 1.045–1.505) and renal dysfunction (per 100-μmol/L increase; P = 0.019, HR; 1.045, CI 1.007–1.084) were associated with appropriate ICD therapy. These factors have not been reported elsewhere. For patients with a borderline indication, these factors can aid in the selection of ICD candidates.

### 4.5 Long-term outcome

Little is known about HF events in ACHD patients with ICDs. In the present study, the incidence of HF was higher than in the previous studies [18]. A high prevalence of complex anatomy can contribute to an increase in the incidence of HF events in our cohort. The annual incidence of MACE was 5.0%/year, which is consistent with previous studies [6,18]. Cruz at al. showed that composite outcomes including death, heart transplant, and hospitalization were related to appropriate ICD therapy [18]. However, there was no association between them in our study. The onset of atrial and ventricular arrhythmias are considered as a marker of disease progression [19,20]. ICD shock-induced myocardial stunning and injury are additional mechanisms for decompensation in already compromised ventricle [21], although, ATP therapy for VT is found to be less deleterious than ICD shock [22]. Termination of VT by ATP in a significant number of patients (87.0%) in our cohort can explain the absence of association between ICD therapy and MACE.

## 5 Limitations

Several limitations must be considered for this study. Firstly, the study was a nonrandomized retrospective analysis of a relatively small number of participants from a single center. There was also heterogeneity in the types of congenital heart disease and the programming of ICD therapy. Data were acquired retrospectively, and the number, interval, and documentation of clinical visits varied between patients. This retrospective study included more patients with moderate-severe cardiac defects than the other studies, which might affect the incidence of ICD therapy and cardiac events. Secondly, before the publication of these guidelines, the decision regarding device implantation was often left to the physician's discretion. Thirdly, we used appropriate therapy implicitly as a surrogate for SCD, however, it is certainly possible that many of these events may have terminated spontaneously [23]. Finally, more extensive studies, in the form of prospective registries, are mandatory to fill in the gaps between guidelines and the real world.

## 6 Conclusion

Implantation of ICDs for preventing SCD following guidelines is reasonable. For patients with a borderline indication, younger age and renal dysfunction may aid in the selection of ICDs candidates.

## Declaration of competing interest

The authors declare that they have no known competing financial interests or personal relationships that could have appeared to influence the work reported in this paper.
