# Supplementary material for: The validity of current implantable cardioverter-defibrillator guidelines in a real-world population of adults with congenital heart disease: A single-center experience
Source: Int J Cardiol Congenit Heart Dis. 2022 Mar 17;8:100355. doi: 10.1016/j.ijcchd.2022.100355 (PMC11657946; doi:10.1016/j.ijcchd.2022.100355)
Supplement: Multimedia component 1 [file mmc1.docx]

Supplement Figure 1.

Supplement Figure 2.

Supplement Figure 3.

Supplement Table 1.

|  | Appropriate ICD therapy (+)  (N=23) | Appropriate ICD therapy (-)  (N=108) | P value |
| --- | --- | --- | --- |
| Age (years) | 34.9±12.9 | 44.5±14.6 | 0.003 |
| Sex (Male) | 18 (78.3%) | 79 (71.8%) | 0.527 |
| Body mass index (kg/m2) | 25.6±4.4 | 27.1±5.8 | 0.915 |
| Secondary prevention (%) | 15 (65.2%) | 47 (42.7%) | 0.049 |
| Class Ⅰ indication (%) | 20 (87.0%) | 64 (59.3%) | 0.010 |
| Complex anatomy (%) | 13 (56.5%) | 48 (43.6%) | 0.259 |
| Biventricular pacing system (CRTD) (%) | 7 (30.4%) | 48 (44.4%) | 0.216 |
| BNP (median) (pg /ml) | 376.9 (160.1-1059) | 249.5 (119.6-529.3) | 0.734 |
| Creatinine (umol /L) | 122.6±153.5 | 94.1±42.2 | 0.114 |
| Albumin (g/dl) | 36.7±4.5 | 39.2±5.1 | 0.062 |
| QRS duration (ms) | 169.8±43.4 | 163.0±38.3 | 0.497 |
| Echocardiogram |  |  |  |
| Systemic ventricular EF (%) | 43.7±16.1 | 39.5±16.1 | 0.281 |
| Subpulmonary ventricular FAC (%) | 32.0±3.0 | 26.0±10.2 | 0.323 |
| Subpulmonary systolic ventricular pressure (mmHg) | 45.9±18.6 | 45.4±13.5 | 0.912 |
| Previous VT ablation (%) | 1 (4.3%) | 8 (7.3%) | 0.612 |
| Heart failure  admission history (%) | 9 (39.1%) | 57 (51.8%) | 0.268 |
| Coronary artery disease (%) | 1 (4.3%) | 6 (5.5%) | 0.829 |
| Previous AF/AT (%) | 5 (41.7%) | 30 (68.2%) | 0.093 |
| Beta blocker (%) | 17 (81.0%) | 83 (76.9%) | 0.680 |
| ACEI/ARB (%) | 8 (38.1%) | 50 (46.3%) | 0.489 |
| Amiodarone (%) | 6 (28.6%) | 35 (32.4%) | 0.730 |

Supplement Table 2.

|  | TOF (n=35) | D-TGA (n=27) | cc TGA (n=22) | AS (n=7) | DOV (n=6) | Ebstein (n=5) | ASD (n=5) | Others (n=24) | P value |
| --- | --- | --- | --- | --- | --- | --- | --- | --- | --- |
| Age (years) | 47.0±15.3 | 37.9±10.1 | 42.5±16.8 | 56.0±10.1 | 43.8±16.6 | 33.8±17.5 | 52.4±17.8 | 38.3±12.2 | 0.010 |
| Sex (Male) | 77.1% | 74.1% | 72.7% | 85.7% | 50.0% | 60.0% | 40.0% | 79.2% | 0.517 |
| Body mass index (kg/m2) | 27.6±6.5 | 25.4±5.1 | 26.8±5.4 | 29.0±5.6 | 31.6±4.1 | 29.2±5.9 | 25.9±7.6 | 25.7±4.0 | 0.311 |
| NYHA Ⅱ-Ⅲ (n, %) | 81.3% | 66.7% | 83.3% | 83.3% | 50.0% | 50.0% | 100.0% | 60.0% | 0.562 |
| Primary prevention (%) | 11 (31.4%) | 15 (55.6%) | 20 (90.9%) | 7 (100.0%) | 1 (16.7%) | 1 (20.0%) | 4 (80.0%) | 10 (41.7%) | 0.001 |
| Secondary prevention (%) | 24 (68.6%) | 12 (44.4%) | 2 (9.1%) | 0 (0.0%) | 5 (83.3%) | 4 (80.0%) | 1 (20.0%) | 14 (58.3%) |  |
| Documented VT | 17 (70.8%) | 9 (75.0%) | 2 (100.0%) | 0 (0.0%) | 5 (100.0%) | 1 (25.0%) | 0 (0.0%) | 5 (35.7%) | 0.028 |
| Documented VF (including cardiac arrest) | 7 (29.2%) | 3 (25.0%) | 0 (0.0%) | 0 (0.0%) | 0 (0.0%) | 3 (75.0%) | 1 (100.0%) | 9 (64.3%) |  |
| Biventricular pacing system (CRT-D) (%) | 21 (60%) | 21 (77.8%) | 5 (22.7%) | 0 (0.0%) | 4 (66.7%) | 4 (80.0%) | 2 (40.0%) | 19 (79.2%) | 0.001 |
| Total follow up periods (month) | 46.2±32.7 | 43.0±26.8 | 37.4±27.6 | 44.7±23.5 | 52.0±22.5 | 28.4±24.2 | 25.0±18.1 | 35.9±28.3 | 0.536 |
| BNP (pg/ml) | 399.0 (182.0-525.4) | 192.0 (124.8-713.5) | 659.4 (182.4-341.2) | 347.7 (97.3-439.1) | NA | NA | NA | 18.6 (105.2-268.1) | 0.875 |
| Creatinine (umol/L) | 97.4±52.3 | 113.7±129.0 | 91.2±23.1 | 131.6±84.6 | 76.4±8.4 | 92.0±28.2 | 89.2±21.2 | 85.3±24.5 | 0.752 |
| Albumin (g/dl) | 38.6±5.4 | 38.6±4.7 | 40.4±5.4 | 41.4±3.6 | 34.6±3.6 | 34.0±2.8 | 34.0±6.1 | 39.2±4.4 | 0.138 |
| QRS duration (ms) | 178.0±34.5 | 152.1±38.4 | 168.1±35.0 | 201.1±31.7 | 186.4±17.3 | 184.7±9.0 | 118.8±36.4 | 144.7±42.2 | 0.001 |
| Echocardiogram |  |  |  |  |  |  |  |  |  |
| Systemic ventricular EF (%) | 43.9±17.0 | 34.2±12.7 | 29.0±8.2 | 27.0±5.6 | 49.7±14.1 | 52.0±13.6 | 39.8±17.0 | 47.4±17.1 | 0.001 |
| Subpumonary venricular FAC (%) | 29.7±8.5 | 36.5±0.7 | 12.3±14.5 | 25 | 24.3±4.6 | 20.0±21.0 | 12 | 21.2±6.7 | 0.053 |
| Subpulmonary systemic ventricular pressure (mmHg) | 46.4±15.1 | 42.8±10.0 | 55.5±15.4 | 38.6±11.9 | 44.8±16.2 | 46.0±14.1 | 39.4±14.2 | 43.6±13.9 | 0.307 |
| EPS before ICD implantation (%) | 20 (57.1%) | 6 (22.2%) | 3 (13.6%) | 1 (14.3%) | 0 (0.0%) | 3 (60.0%) | 0 (0.0%) | 4 (3.1%) | 0.001 |
| VT/VF induction (%) | 15 (75.0%) | 5 (83.3%) | 1 (33.3%) | 0 (0.0%) | NA | 3 (100.0%) | NA | 0 (0.0%) | 0.016 |
| Previous VT ablation (%) | 6 (17.1%) | 2 (7.4%) | 0 (0.0%) | 0 (0.0%) | 0 (0.0%) | 1 (20.0%) | 0 (0.0%) | 0 (0.0%) | 0.105 |
| Heart failure admission history (%) | 13 (37.1%) | 13 (48.1%) | 17 (77.3%) | 6 (85.7%) | 2 (33.3%) | 1 (20.0%) | 3 (60.0%) | 10 (41.7%) | 0.027 |
| Coronary artery disease (%) | 2 (5.7%) | 1 (3.7%) | 2 (9.1%) | 1 (14.3%) | 0 (0.0%) | 0 (0.0%) | 0 (0.0%) | 1 (4.2%) | 0.899 |
| Beta blocker (%) | 24 (72.7%) | 22 (81.5) | 17 (81.0%) | 5 (71.4%) | 4 (80.0%) | 4 (80.0%) | 5 (100.0%) | 18 (75.0%) | 0.922 |
| ACEI /ARB (%) | 8 (24.2%) | 10 (37.0%) | 16 (76.2%) | 4 (57.1%) | 2 (40.0%) | 2 (40.0%) | 2 (40.0%) | 13 (54.2%) | 0.024 |
| Amiodarone (%) | 10 (30.3%) | 10 (37.0%) | 6 (28.6%) | 3 (42.9%) | 4 (80.0%) | 1 (20.0%) | 1 (20.0%) | 4 (16.7%) | 0.218 |

Supplement Table 3.

|  | Univariate analysis | | | Multivariate analysis | | |
| --- | --- | --- | --- | --- | --- | --- |
|  | HR | 95% CI | P value | HR | 95% CI | P value |
| Age (10-years decrease) | 1.172 | 1.023-1.344 | 0.022 | 1.254 | 1.045-1.505 | 0.015 |
| Sex (Male) | 1.237 | 0.458-3.340 | 0.675 |  |  |  |
| Body mass index (kg/m2) | 0.951 | 0.865-1.046 | 0.300 |  |  |  |
| PACES/HRS ClassⅠ(or EHRA recommended/indicated) indication | 3.790 | 1.121-12.81 | 0.031 | 6.488 | 0.828-50.84 | 0.075 |
| Secondary prevention | 1.981 | 0.830-4.726 | 0.123 |  |  |  |
| Complex anatomy (vs moderate and simple) | 0.595 | 0.260-1.362 | 0.219 |  |  |  |
| Total follow up periods (/month) | 0.996 | 0.980-1.013 | 0.642 |  |  |  |
| Creatinine (umol/L) (100-umol /L increase) | 1.528 | 1.032-2.262 | 0.034 | 1.555 | 1.076-2.247 | 0.019 |
| Albumin (umol/L)  (10 umol /L increase) | 0.392 | 0.160-0.959 | 0.040 |  |  |  |
| BNP (pg/ml) | 1.053 | 0.303-3.661 | 0.936 |  |  |  |
| QRS duration (ms) | 1.004 | 0.992-1.017 | 0.516 |  |  |  |
| Systemic ventricular EF (%) | 1.010 | 0.860-1.036 | 0.403 |  |  |  |
| Subpulmonary ventricular FAC (%) | 1.061 | 0.917-1.229 | 0.426 |  |  |  |
| Subpulmonary systolic ventricular pressure (mmHg) | 1.005 | 0.970-1.041 | 0.775 |  |  |  |
| Previous VT ablation | 1.680 | 0.226-12.48 | 0.612 |  |  |  |
| Previous AF/AT | 0.357 | 0.113-1.125 | 0.079 |  |  |  |
| Beta blocker | 0.736 | 0.247-2.197 | 0.583 |  |  |  |
| Amiodarone | 1.199 | 0.464-3.095 | 0.708 |  |  |  |
| ACEI/ARB | 1.283 | 0.530-1.283 | 0.581 |  |  |  |
